# M1 polarization enhances the antitumor activity of chimeric antigen receptor macrophages in solid tumors

**DOI:** 10.1186/s12967-023-04061-2

**Published:** 2023-03-28

**Authors:** Yi Huo, Han Zhang, Longqi Sa, Wenjing Zheng, Yang He, Haohan Lyu, Mengjie Sun, Lingling Zhang, Lequn Shan, Angang Yang, Tao Wang

**Affiliations:** 1 State Key Laboratory of Cancer Biology, Department of Immunology, Air Force Medical University, Xi’an, Shaanxi China; 2 State Key Laboratory of Cancer Biology, Department of Medical Genetics and Developmental Biology, Air Force Medical University, Xi’an, Shaanxi China; 3 Department of Laboratory Medicine, 941 Hospital of Joint Logistics Support Force of PLA, Xining, Qinghai China; 4 Department of Spine Surgery, Honghui Hospital, Xi’an Jiaotong University, Xi’an, Shaanxi China; 5 The Second Clinical Medical College of Lanzhou University, Lanzhou University, Lanzhou, Gansu China

**Keywords:** Chimeric antigen receptor, Macrophages, CAR-M, M1 polarization, HER2, Cancer immunotherapy

## Abstract

**Background:**

Chimeric antigen receptor macrophage (CAR-M) therapy is a novel cancer immunotherapy approach that integrates CAR structure and macrophage functions. CAR-M therapy has shown unique and impressive antitumor effects in immunotherapy for solid tumors. However, the polarization state of macrophages can affect the antitumor effect of CAR-M. We hypothesized that the antitumor activity of CAR-Ms may be further improved after inducing M1-type polarization.

**Methods:**

In this report, we constructed a novel HER2-targeting CAR-M, which was composed of humanized anti-HER2 scFv, CD28 hinge region and FcγRI transmembrane domain and intracellular domain. Phagocytosis, tumor-killing capacities, and cytokine release of CAR-Ms were detected with or without M1-polarization pretreatment. Several syngeneic tumor models were used to monitor the in vivo antitumor activity of M1-polarized CAR-Ms.

**Results:**

After polarization with LPS combined with interferon-γ in vitro, we found that the phagocytic and tumor-killing capacities of CAR-Ms against target cells were significantly enhanced. The expression of costimulatory molecules and proinflammatory cytokines was also significantly increased after polarization. By establishing several syngeneic tumor models in vivo, we also demonstrated that infusing polarized M1-type CAR-Ms could effectively suppress tumor progression and prolong the survival of tumor-bearing mice with enhanced cytotoxicity.

**Conclusions:**

We demonstrated that our novel CAR-M can effectively eliminate HER2-positive tumor cells both in vitro and in vivo, and M1 polarization significantly enhanced the antitumor ability of CAR-M, resulting in a stronger therapeutic effect in solid cancer immunotherapy.

**Supplementary Information:**

The online version contains supplementary material available at 10.1186/s12967-023-04061-2.

## Introduction

Cancer immunotherapy represents a promising approach for the treatment of malignancies. Cancer immunotherapy approaches mainly include immune checkpoint blockade, adoptive cell therapy, cancer vaccines and oncolytic viruses. Adoptive cell therapy effectively kills cancer cells by redirecting specific immune cells to neighboring cancer cells to exert cytotoxic effects. Chimeric antigen receptor T (CAR-T) cells have shown efficacy in the treatment of certain hematologic malignancies [1, 2]. Several CAR-T-cell therapy products targeting CD19 and BCMA have been approved by the FDA for use in the clinic, but the application of CAR-T cells to solid malignancies faces great challenges [3]. Poor trafficking and infiltration, the immunosuppressive tumor microenvironment, and the heterogeneity of tumor antigens are key factors limiting CAR-T-cell function in solid tumors [4]. These limitations have also prompted researchers to continue to explore other immune cells as alternative therapeutic tools [5].

As essential players in innate immunity, macrophages play critical roles in host defense and tissue homeostasis [6]. Owing to their excellent trafficking ability and natural penetration capacity, unique immunomodulatory function, and phagocytosis and killing activity, engineered macrophages have been exploited to establish novel cellular immunotherapy approaches for malignancies [7]. Engineered macrophages have emerged as alternative candidates for CAR-based therapies. Transducing macrophages with CAR has been demonstrated to induce effective antitumor effects in both preclinical models and clinical trials [8–11]. Chimeric antigen receptor-macrophages (CAR-Ms) specifically recognize cancer cells for phagocytosis. In addition to direct phagocytosis and killing, CAR-Ms can further activate the host adaptive immune response to generate synergistic antitumor effects via antigen presentation and epitope spreading [8].

The phenotypes and functions of macrophages are plastic and switch dynamically in response to cytokines, pathogen-associated molecular patterns and environmental signals [6, 12]. Tumor-associated macrophages (TAMs) are a complex and heterogeneous population of cells within the tumor microenvironment (TME), and they include antitumor (M1-like) macrophages with tumoricidal activity and protumor (M2-like) macrophages with immunosuppressive functions that support tumor growth and promote metastasis [13, 14]. Although M1-like macrophages have potential antitumor effects, most TAMs behave as M2-like macrophages and play a protumorigenic role. Thus, TAMs have become a potential target for cancer immunotherapy. The high plasticity of macrophages has led to attempts to reprogram protumor macrophages toward antitumor macrophages, which is a promising immunotherapeutic strategy based on macrophage function [13]. Accurately defining macrophage characteristics and subpopulations is essential for advancing macrophage-based immunotherapies.

Further improving CAR-M function may lead to enhanced immunotherapeutic efficacy and the development of potent treatments for solid tumors. Based on the theory of macrophage polarization, we hypothesized that polarizing M1-type CAR-Ms might enhance the proinflammatory phenotype of macrophages and result in enhanced antitumor therapeutic effects, with the potential to prime the adaptive antitumor immune response. In this study, we artificially polarized CAR-Ms and evaluated their antitumor activity both in vitro and in vivo. We found that although both CAR-Ms and M1-polarized CAR-Ms could suppress the progression of solid tumors, M1-polarized CAR-Ms exerted more potent therapeutic effects than CAR-Ms. We performed a proof-of-principle experiment in murine tumor models by applying M1-polarized CAR-Ms for immunotherapy.

## Materials and methods

### Mice

Wild-type six- to eight-week-old female C57BL/6 mice were purchased from the Experimental Animal Facility of the Air Force Medical University, Xi’an, China, and were bred in experimental animal facilities under specific pathogen-free conditions. All animal studies in vivo were conducted in compliance with institutional guidelines for the humane treatment of animals and were approved by the Institutional Animal Care and Use Committee of the Air Force Medical University.

### Cell lines

The cell lines MC38, B16F10, ID8, 293 T and J774A.1 were purchased from Procell Life Science & Technology Co., Ltd., Wuhan, China. The murine tumor cell lines MC38 and B16F10 were cultured in RPMI-1640 medium. The ID8, 293 T and J774A.1 cell lines were cultured in high-glucose DMEM. All media were supplemented with 10% FBS (Gibco), 100 µM nonessential amino acids (Gibco), 2 mM L-glutamine (Gibco), and 1% penicillin‒streptomycin (Gibco). The cells were cultured in an atmosphere of 5% CO_2_ at 37 °C.

The murine tumor cell lines MC38 and ID8 were first transduced with a lentiviral vector encoding luciferase and mCherry. After sorting, the luciferase- and mCherry-positive murine cell lines were further transduced with lentivirus encoding a truncated human HER2 gene that was expressed on the cell surface but was unable to drive intercellular signal transduction. Flow cytometry was performed to sort cells with high HER2 expression, which served as target cells in this study.

Bone marrow-derived macrophages (BMDMs) were differentiated from bone marrow cells stimulated with 25 ng/ml M-CSF (PeproTech) for 7 days. Primary macrophages were cultured in DMEM supplemented with 10% FBS.

### Tumor models in vivo

Schematic diagrams of the tumor xenograft models are shown in the first panel of each relevant figure. To establish a syngeneic tumor model, tumor cells in logarithmic growth phase were harvested and suspended in cold PBS for inoculation.

Briefly, for the intraperitoneal tumor-bearing model, 2*10^6^ Luc^+^ ID8-HER2 cells were inoculated intraperitoneally. Mice were intraperitoneally injected with macrophages at 14 days and 21 days post inoculation. Tumor burden was monitored with an IVIS system, and survival was assessed daily.

For the subcutaneous tumor model, 2*10^5^ B16F10-HER2 melanoma cells were inoculated subcutaneously in the right flank of C57BL/6 mice. When the average tumor volume reached almost 50 mm^3^ on day 7 after inoculation, the mice were randomly divided into four groups and received an intravenous infusion of engineered macrophages. Tumor volumes were measured twice weekly with an electronic caliper and were calculated according to the formula (L × W × W)/2. Mice were weighed every three days. Tumor-bearing mice were euthanized once the tumor volume reached 2000 mm^3^.

For the lung metastasis model, 1*10^6^ B16F10-HER2 cells were inoculated intravenously. Mice were randomized on day 7 after B16F10-HER2 cell inoculation. The infusion was split into repeated intravenous injections on days 7 and 10 to achieve the required dose. On day 14, the lungs were harvested and analysed.

Bioluminescence imaging was performed weekly using the in vivo Imaging System (PerkinElmer), and data analysis was conducted using LivingImage v4.3.1 (Caliper Life Sciences).

### Plasmid construction and lentivirus production

The anti-HER2 CAR sequence was synthesized and cloned into the lentiviral vector backbone pRRLSIN.cPPT.RFPL4b. All cloning steps were validated through restriction enzyme digest reactions, and the whole constructs were sequenced. The transfer plasmid encoding CAR, the viral envelope plasmid and the packaging plasmid were cotransfected into HEK293T cells using PEI transfection reagent (AC04L091, Shanghai Life-iLab Biotech). The lentivirus-containing supernatants from transfected 293 T cells were collected at 48 h and 72 h after transfection and filtered through a 0.45 µm filter (Millipore). The filtered supernatants were mixed with PEG6000 overnight at 4 °C and then concentrated by centrifugation at 4500 × g for 30 min at 4 °C. Macrophages were transduced with lentivirus at an MOI of 50 with 10 µg/ml polybrene.

### Flow cytometry

Cells were prepared in flow cytometry buffer and stained at 4 °C for 30 min with appropriate fluorescence-conjugated antibodies. The antibodies used for flow cytometry analyses are listed in Additional file 1: Table S1. For each test, 1*10^6^ cells were collected for analysis. Flow cytometry experiments were performed on a BD FACS Canto™ II (BD Pharmingen, San Diego, CA, USA) and were analyzed using FlowJo software (TreeStar, Ashland, OR, USA).

Anti-HER2-CAR expression in primary murine macrophages was detected with recombinant HER2 protein conjugated with a His tag. A two-step staining protocol was performed: human HER2 protein containing a His tag (10004-H08H-100, Sino Biological) was incubated with the macrophages for 2 h at 4 °C, followed by staining with APC conjugated antibody that recognized the His tag portion of the recombinant HER2 protein for the flow cytometry assay.

### Flow cytometry-based phagocytosis assay

A total of 1*10^5^ GFP-Ms or anti-HER2 CAR-Ms (treated with 4 µg/ml puromycin for 48 h after lentiviral transduction to screen transduced cells) were cocultured with 2*10^4^ mCherry-expressing target cells with cell‒cell contact for 1 h at 37 °C. After coculture, total cells were harvested, washed with PBS and then analyzed by flow cytometry. The percentage of mCherry-expressing cells within the GFP-positive macrophage population represented the target cells phagocytosed by macrophages.

### In vitro* cytotoxicity assay*

Luciferase-expressing MC38-HER2 cells were used as targets in luciferase-based killing assays. For the coculture killing assay in vitro, macrophages were plated at a density of 2*10^4^ per well in a white 96-well microplate and allowed to adhere for 6 h. Luc^+^ MC38-HER2 cells were placed at different E:T ratios and cocultured with macrophages for 24 h or 48 h at 37 °C. Bioluminescence was measured with an IVIS Spectrum (PerkinElmer). Specific lysis was calculated on the basis of the luciferase signal relative to that of tumor cells alone using the following formula. Three independent experiments were performed.

$${\text{Lysis}}\,\left( \% \right)\, = \,[({\text{Sample signal}} - {\text{Tumor only signal}})\left] / \right[({\text{Background signal}} - {\text{Tumor only signal}})]*{1}00$$

### Microscopy

1*10^5^ macrophages were plated in 24-well plates. mCherry^+^ MC38-HER2 cells were added and cocultured at 37 °C for the indicated times. The phagocytic events in three random fields per well were averaged for triplicate wells.

### Macrophage polarization

For M1-type macrophage polarization, bone marrow-derived macrophages were stimulated with 20 ng/ml recombinant mouse interferon-γ (50709-MNAH, Sino Biological) and 50 ng/ml lipopolysaccharide (L5293, Sigma‒Aldrich Trading) for 24 h. Polarized macrophages were washed with PBS before infusion.

### Real-time PCR

Total RNA was extracted using a MiniBEST Universal RNA Extraction Kit (9767, Takara). 1 μg of total RNA was reverse transcribed using the PrimeScript Reverse Transcriptase Kit (Takara). For real-time PCR, template cDNA, primers, and TB Green Fast qPCR Mix (Takara) were mixed according to the manufacturer’s instructions. The housekeeping gene GAPDH was used for normalization of the samples. The relative mRNA levels (fold changes) of each gene among the different samples were quantified using the comparative 2^−△△Ct^ method. The primers were synthesized by Tsingke Biotechnology.

### Cytokine ELISA

Mouse IL-1β (VAL601, Bio-Techne China), IL-12p70 (VAL606, Bio-Techne China), and TNF-α (VAL609, Bio-Techne China) concentrations in cell culture supernatants were assayed using commercial ELISA kits according to the manufacturer’s instructions.

### Statistics

Statistical analysis was performed with GraphPad Prism 9.4 (GraphPad). The statistical significance of differences between different groups was calculated using ANOVA with a multiple comparisons test. The statistical significance of Kaplan‒Meier survival was calculated via the log-rank Mantel‒Cox test. For all statistics in this study, * indicates P < 0.05, ** indicates P < 0.01, *** indicates P < 0.001.

## Results

### Anti-HER2 CAR-Ms specifically target HER2-positive cancer cells

**Fig. 1:**
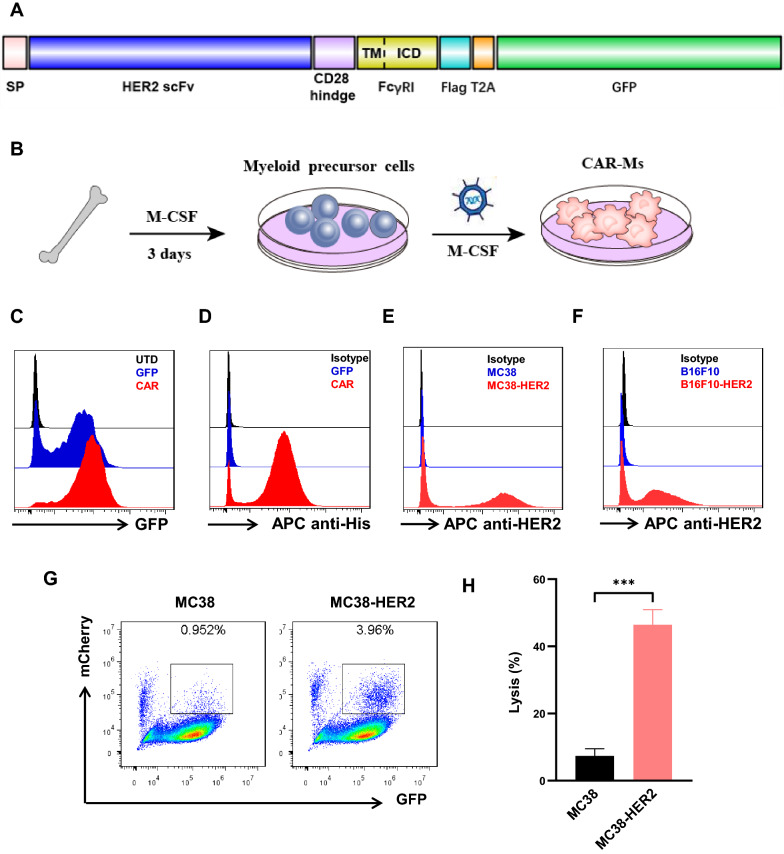
Anti-HER2 CAR-Ms specifically target HER2-positive cancer cells. **A** A diagram of the anti-HER2-CAR construct. **B** Overview of primary macrophage differentiation from bone marrow and the lentivirus transduction protocol. **C** Representative flow cytometry analysis of the CAR transduction frequency in lentivirus-transduced primary macrophages after 72 h. **D** Representative flow cytometry analysis of CAR expression on macrophages after lentivirus transduction, with recombinant HER2 protein binding and staining with an APC-conjugated anti-His tag antibody. **E–F** Representative flow cytometry analysis of HER2 expression in sorted cells of the murine tumor cell lines MC38 (**E**) and B16F10 (**F**) after stable transduction with the truncated HER2 lentivirus. **G** Flow cytometry-based phagocytosis of mCherry^+^ MC38-wild type or mCherry^+^ MC38-HER2 target cells by anti-HER2 CAR-Ms at an E:T = 5:1 ratio. The double-positive cell population represents the target cells engulfed by macrophages. **H** Luciferase-based killing assay of Luc^+^ MC38-wild type or Luc^+^ MC38-HER2 target cells by anti-HER2 CAR-Ms at an E:T = 5:1 ratio in vitro. ***, *p* < 0.001

We designed novel anti-HER2 CAR-Ms (hereafter referred to as CAR-Ms). The extracellular antigen recognition domain was derived from a humanized single-chain antibody variable region (scFv) and recognized the HER2 antigen with high affinity, as reported previously [15]. The hinge domain was part of the murine CD28 extracellular sequence, and the transmembrane and intracellular signal domains were from the Fc receptor I common γ subunit, a canonical signaling molecule that triggers engulfment in macrophages (Fig. 1A). The intracellular domain of FcγRI contains immunoreceptor tyrosine-based activation motifs (ITAMs), which promote phagocytosis and activation of macrophages. Our CAR constructs also contain green fluorescent protein (GFP) to serve as a marker for transduction. Control macrophages were transduced with GFP-empty vector and were named GFP-Ms. Anti-HER2 CAR sequences were synthesized and cloned into the lentiviral plasmid backbone pRRLSIN.cPPT. RFPL4b to construct the lentiviral vector. Using a third-generation lentiviral packaging system, the virus was produced in 293 T cells. The lentiviral supernatants were concentrated and added to murine BMDMs for transduction (Fig. 1B). Primary BMDMs exhibited approximately 95% purity, as indicated by CD11b and F4/80 staining via flow cytometry (Additional file 1: Fig. S1). Flow cytometry analysis demonstrated that lentiviral transduction led to GFP expression in primary macrophages with high efficiency (Fig. 1C). Over 80% of macrophages were GFP-positive at 72 h after lentiviral infection in both the GFP-M and CAR-M groups, indicating high CAR transduction efficiency. To further confirm CAR expression on transduced macrophages, recombinant HER2 protein containing a His tag was incubated with macrophages and then detected by flow cytometry with anti-His-APC antibodies. The flow cytometry results showed that CAR-positive macrophages accounted for more than 80% of cells in the CAR-M group (Fig. 1D), which was basically consistent with the proportion of GFP-positive macrophages detected. These results indicated that CAR could be efficiently transduced into macrophages.

To determine whether CAR-Ms specifically recognize HER2-positive tumor cells, we constructed a lentiviral vector that expressed a truncated human *ERBB2* gene. This truncated gene encoded the extracellular and transmembrane domains of the human HER2 protein, and when it was expressed on HER2-negative cells, it could endow those cells with epitopes from the HER2 antigen while avoiding the effects of HER2 signaling. MC38 murine colorectal adenocarcinoma cells and B16F10 melanoma cells were first infected with lentivirus encoding luciferase and mCherry. These cells were then further infected with lentivirus expressing truncated human HER2, and cells stably expressing high levels of human truncated HER2 were detected by flow cytometry (Fig. 1E, F). We next incubated CAR-Ms with either Luc^+^ MC38 cells or HER2-expressing Luc^+^ MC38-HER2 cells. We found that CAR-Ms engulfed tumor cells in an antigen-specific manner (Fig. 1G) and that HER2-expressing tumor cells were more likely to be killed by CAR-Ms (Fig. 1H). These data demonstrated that CAR-Ms were able to specifically target HER2-positive cancer cells.

### M1 polarization enhances CAR-M phagocytosis and cytotoxic effects

**Fig. 2:**
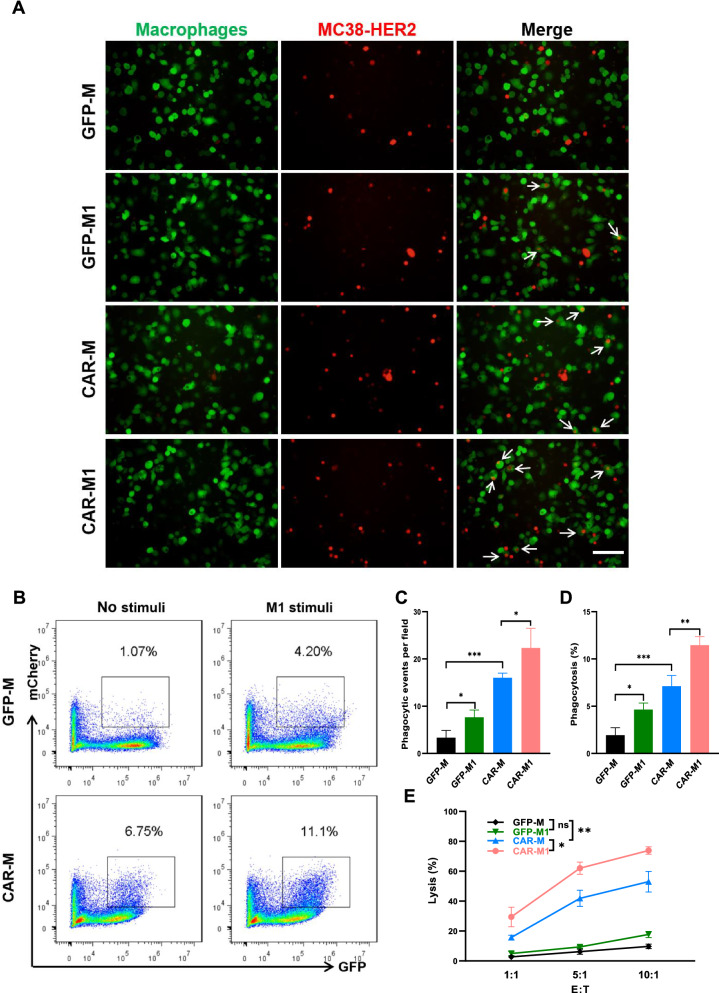
CAR-M-mediated phagocytosis and cytotoxicity were enhanced after M1-type polarization. **A** Representative fluorescence microscopy images showing GFP-positive macrophage-mediated phagocytosis after 4 h of coincubation with mCherry^+^ MC38-HER2 cells, scale bar: 50 µm. **B** Representative flow cytometry analysis of the phagocytosis of mCherry^+^ MC38-HER2 target cells by control or M1-polarized macrophages at an E:T = 5:1 ratio. The double-positive cell population represents the target cells engulfed by macrophages. **C** Quantitative analysis of the data from (**A**). Three random fields of view were assessed per replicate, and data are represented as the mean ± SEM. of three independent experiments. **D** Statistical analysis of the percentage of phagocytosis by flow cytometry. Three independent experiments were performed. Data are shown as the mean ± SEM. **E** Cytotoxicity was evaluated by a luciferase-based killing assay with Luc^+^ MC38-HER2 target cells and macrophages after 24 h of coculture at different E:T ratios in vitro. Data are represented as the mean ± SEM. of three independent experiments. *, *p* < 0.05; **, *p* < 0.01; ***, *p* < 0.001

To assess phagocytosis in macrophages, GFP-Ms and CAR-Ms were coincubated with mCherry-expressing MC38-HER2 cells. The results of fluorescence microscopy showed that although GFP-Ms could hardly engulf tumor cells, CAR-Ms had a significant phagocytic effect, and the phagocytosis capacity of both GFP-Ms and CAR-Ms was significantly improved after M1 polarization (Fig. 2A, C). In flow cytometry analysis, we obtained the same results as fluorescence microscopy imaging. The double-positive cells of mCherry and GFP represented macrophages that had engulfed tumor cells in the flow cytometry-based phagocytosis assay. We found that only approximately 1% of GFP-Ms engulfed tumor cells, while the proportion of phagocytosis increased to 4.2% after M1 polarization. Similarly, the proportion of tumor cell phagocytosis of CAR-Ms increased significantly from 6.75% to 11.7% after M1 polarization (Fig. 2B, D). This result was consistent with the phagocytosis of HER2-positive tumor cells by CAR-Ms derived from the murine J774A.1 macrophage cell line (Additional file 1: Fig. S2A, B). Overall, these data clearly suggested that CAR-Ms can efficiently engulf HER2-positive tumor cells and that M1 polarization can further enhance this phagocytic effect.

We next evaluated the cytotoxic effect of CAR-Ms in vitro through a luciferase-based bioluminescence assay. Our data showed that the cytotoxicity of CAR-Ms was associated with the effector-to-target ratio (E:T). With a gradual increase in the E:T ratio, the killing activity of CAR-Ms increased accordingly (Fig. 2E). When the E:T ratio reached 10:1, the killing effect of CAR-Ms on Luc^+^ MC38-HER2 tumor cells reached approximately 50% after coculture of the target cells with CAR-Ms for 24 h. As expected, the killing capacity of CAR-Ms was further enhanced after M1 polarization, resulting in the elimination of approximately 70% of cancer cells in 24 h. In contrast, macrophages transduced with empty GFP vector showed neither potent tumor-killing effects nor significant dose-dependent activity (Fig. 2E). CAR-mediated killing potential was also evaluated using the J774A.1 macrophage cell line, and a time- and dose-dependent tendency was observed (Additioanl file 1: Fig. S3A-D). In addition, we used YOYO3, a far-red fluorescent dye, to label dead tumor cells and found that coculture of CAR-Ms and M1-polarized CAR-Ms with HER2-positive tumor cells resulted in obvious YOYO3-positive dead cells, and there were more dead tumor cells in the M1-polarized CAR-M group (Additioanl file 1: Fig. S3E, F), indicating that CAR-Ms exhibit stronger antitumor activity after M1 polarization.

### M1 polarization enhances CAR-M proinflammatory cytokine secretion

**Fig. 3:**
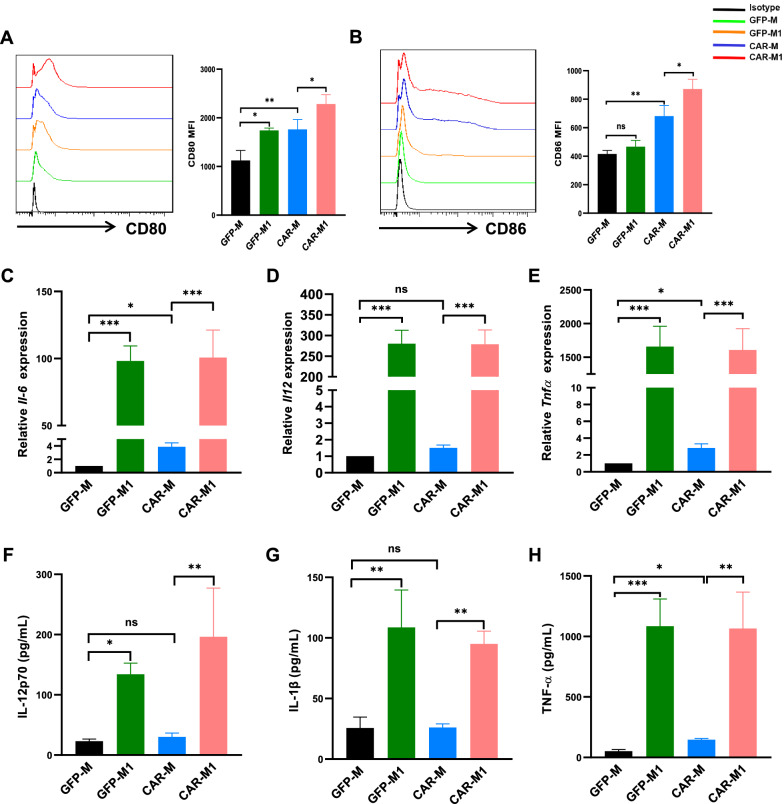
M1-polarized CAR-Ms exhibited stronger proinflammatory phenotypes than CAR-Ms. **A**-**B** Flow cytometry analysis and quantitative analysis of the mean fluorescence intensity (MFI) of the activation markers CD80 (**A**) and CD86 (**B**) on macrophages after coculture with MC38-HER2 target cells. **C**-**E** Normalized gene expression analysis of the proinflammatory cytokines Il-6 (**C**), Il-12 (**D**), and Tnf-α (**E**) by qRT‒PCR. **F**–**H** ELISA to detect secreted proinflammatory cytokines, including IL-12p70 (**F**), IL-1β (**G**) and TNF-α (**H**), in the supernatants of cocultures of macrophages with target cells after 24 h. *, *p* < 0.05; **, *p* < 0.01; ***, *p* < 0.001

To assess the phenotype of CAR-Ms, we examined the expression of the costimulatory ligands CD80 and CD86 by flow cytometry. The results showed that the expression levels of both CD80 and CD86 increased on macrophages after coculturing CAR-Ms with MC38-HER2 cells (Fig. 3A, B), suggesting an enhanced antigen presentation function and activated status of CAR-Ms. In addition, the expression of CD80 and CD86 was further increased when the macrophages were stimulated with LPS and IFN-γ before coculture with HER2-positive cancer cells in both the GFP-M and CAR-M groups (Fig. 3A, B). These results demonstrated that when macrophages were pretreated with M1 stimuli, CAR-Ms obtained stronger antigen presenting ability.

We then asked whether M1 polarization could induce higher proinflammatory gene expression in CAR-Ms. Klichinsky and his colleagues described an adenoviral-based strategy to manufacture CAR-M with primary human monocytes and macrophages. They found that CAR-Ms generated with Ad5f35 were shown to eliminate tumor cells more effectively than the control in vitro and in vivo. Notably, transduction of macrophages with Ad5f35 led to the induction of a durable M1 phenotype [8]. However, whereas CD46, the cellular receptor of Ad5f35, is ubiquitously expressed in humans, expression of mouse CD46 is limited to the testes [16]. Therefore, Ad5f35 is not effective in infecting murine-derived cells. In this study, we established a lentivirus-based strategy for mouse macrophage infection. We found that lentiviral transduction did not result in a significant change in the expression of proinflammatory cytokine genes (Additional file 1: Fig. S4A) and costimulatory molecules in either GFP-Ms or CAR-Ms (Additional file 1: Fig. S4B, C). However, the qPCR results showed that although the increase in *Il-12* was not statistically significant, the expression of the proinflammatory cytokines *Il-6* and *Tnf-α* was significantly higher in the CAR-M group than in the GFP-M group, and both the GFP-M and CAR-M groups exhibited increased *Il-6, Il-12* and *Tnf-α* expression after coculture of engineered macrophages with MC38-HER2 cells (Fig. 3C–E), demonstrating that the proinflammatory phenotype was triggered by the interaction between macrophages and tumor cells rather than by lentiviral infection. More importantly, the elevated expression of IL-12p70, IL-1β and TNF-α in M1-polarized macrophages was further confirmed by ELISA (Fig. 3F–H), indicating that the proinflammatory phenotype of CAR-Ms was significantly enhanced after M1 polarization.

### M1 polarization enhances the antitumor effect of CAR-Ms in an intraperitoneal tumor-bearing model of ovarian cancer

**Fig. 4:**
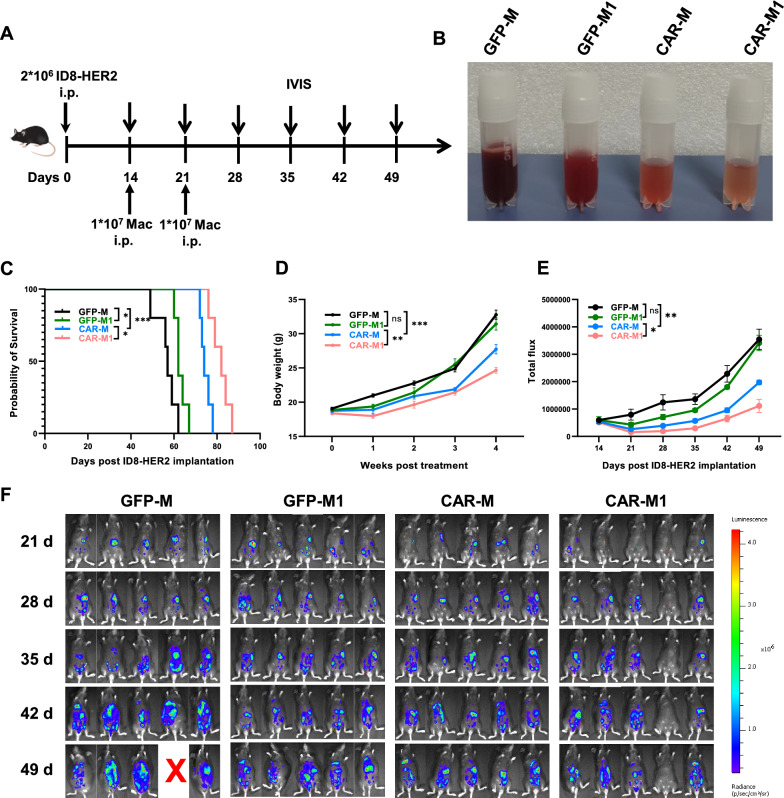
Local administration of polarized CAR-Ms suppressed the progression of ovarian cancer in vivo. **A** Schematic illustration of the experimental design for the ovarian cancer model. **B** The appearance of ascites/peritoneal lavage obtained from tumor-bearing mice after intraperitoneal administration. **C** Kaplan‒Meier survival curve of tumor-bearing mice treated with GFP-M, GFP-M1, CAR-M, and CAR-M1 after ID8-HER2 tumor cell inoculation. **D** Tumor burden assessed by body weight after the mice received GFP-M, GFP-M1, CAR-M, and CAR-M1 treatment. **E** Statistical analysis of bioluminescence imaging. Data are presented as the mean ± S.D. **F** Tumor burden was monitored by bioluminescence imaging on the indicated days after treatment. *, *p* < 0.05; **, *p* < 0.01; ***, *p* < 0.001

To evaluate the antitumor effects of CAR-Ms in vivo, we first established an ovarian cancer intraperitoneal tumor-bearing model in mice (Fig. 4A). After the Luc^+^ ID8-HER2 cells were inoculated intraperitoneally, the mice were intraperitoneally injected with macrophages at 14 days and 21 days post inoculation. We found that compared with GFP-M administration, CAR-M treatment significantly suppressed the progression of ovarian cancer. In addition, although M1-polarized GFP-Ms could prolong the survival of ID8-HER2 tumor-bearing mice, M1-polarized CAR-Ms showed the strongest tumor inhibition effect by effectively reducing the tumor burden and extending the overall survival of tumor-bearing mice (Fig. 4C, E, F). Importantly, with the proliferation of ovarian cancer cells in the peritoneal cavity, the mice in the GFP control groups gradually became visibly sick and began to develop bloody ascites in the abdominal cavity, while mice in the CAR group developed ascites later and significantly less, and mice in the M1-polarized CAR group showed the least bloody ascites accumulation (Fig. 4B). In addition, although all tumor-bearing mice gained weight due to ascites six weeks after tumor cell inoculation, the weight gain in the CAR-M group was significantly smaller than that in the control GFP group (Fig. 4D). The above results suggested that local injection of engineered CAR-Ms, especially M1-polarized CAR-Ms, into the peritoneal cavity could effectively suppress the progression of intraperitoneal ovarian cancer.

### M1-polarized CAR-Ms exhibit a stronger tumor rejection effect in a subcutaneous tumor model

**Fig. 5:**
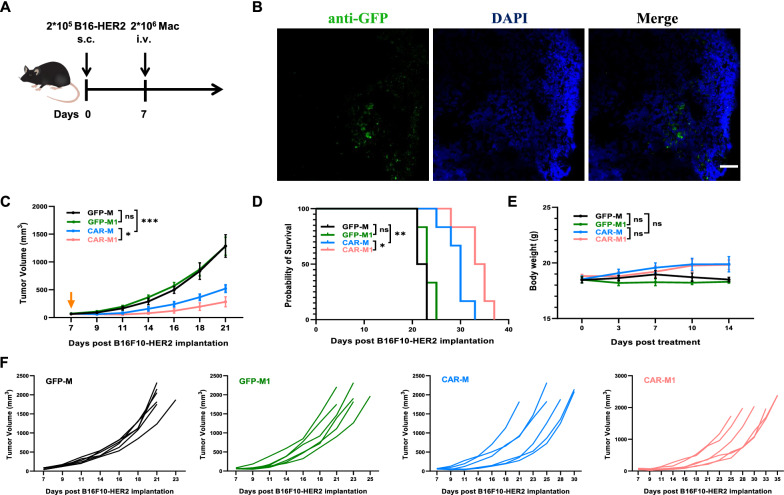
Systemic administration of polarized CAR-Ms induced efficient antitumor effects in a B16F10 melanoma model. **A** An illustration of the experimental design. **B** Representative fluorescent images of melanoma tissue stained with anti-GFP to detect the infiltration of GFP-positive anti-HER2 CAR-Ms, scale bar: 100 μm. **C** Tumor growth of C57 mice bearing subcutaneous B16F10-HER2 melanoma tumors in the GFP-M (black), GFP-M1 (green), CAR-M (blue), and CAR-M1 (red) treatment groups. The average tumor volume per treatment group is presented as the mean ± S.D. **D** Kaplan‒Meier survival curve for (**C**) mice bearing B16F10-HER2 cells after receiving GFP-Ms, GFP-M1s, CAR-Ms, and CAR-M1s (n = 6 mice/group). **E** Body weight changes in tumor-bearing mice posttreatment. The data are shown as the mean ± S.D. **F** Tumor growth of individual mice from (**C**). Each line represents an individual mouse. *, *p* < 0.05; **, *p* < 0.01; ***, *p* < 0.001

To further investigate whether CAR-Ms could elicit effective antitumor immunity, we evaluated the therapeutic effect of systemic CAR-M infusion on tumor progression in a syngeneic subcutaneous melanoma model. Mice received a single dose of macrophages via intravenous injection 1 week after B16F10-HER2 cell implantation (Fig. 5A). As early as 24 h after injection, GFP-positive CAR-M cells were detected in tumor tissue (Fig. 5B). We found that mice adoptively transferred with GFP-M or GFP-M1 could not suppress tumor growth. However, there was a significant reduction in tumor burden in the CAR-M and CAR-M1 groups, and the most potent antitumor effect was observed in the CAR-M1 treatment group (Fig. 5C, F). Although most tumor-bearing mice eventually showed progression, a single i.v. administration of CAR-M1 led to a significantly prolonged overall survival time (Fig. 5D). Overall, these results indicated that intravenous infusion of M1-polarized CAR-Ms led to enhanced antitumor activity in the B16F10 melanoma model.

To evaluate the safety of CAR-Ms for immunotherapy, we monitored toxic reactions, including body weight changes, in tumor-bearing mice posttreatment and performed histopathological analysis of major organs at the endpoints of the experiment. No significant difference in body weight changes was observed among tumor-bearing mice from all treatment groups (Fig. 5E). HE staining analysis of major organs, including the lungs, liver, kidney, and spleen, showed no significant abnormalities in the GFP-M treatment groups or in the CAR-M and M1-polarized CAR-M treatment groups (Additional file 1: Fig. S5A). These results demonstrated that CAR-M treatment was safe and feasible, and no significant toxic reactions associated with treatment were observed in our study.

### M1-polarized CAR-Ms inhibit tumor progression in a lung metastasis model

**Fig. 6:**
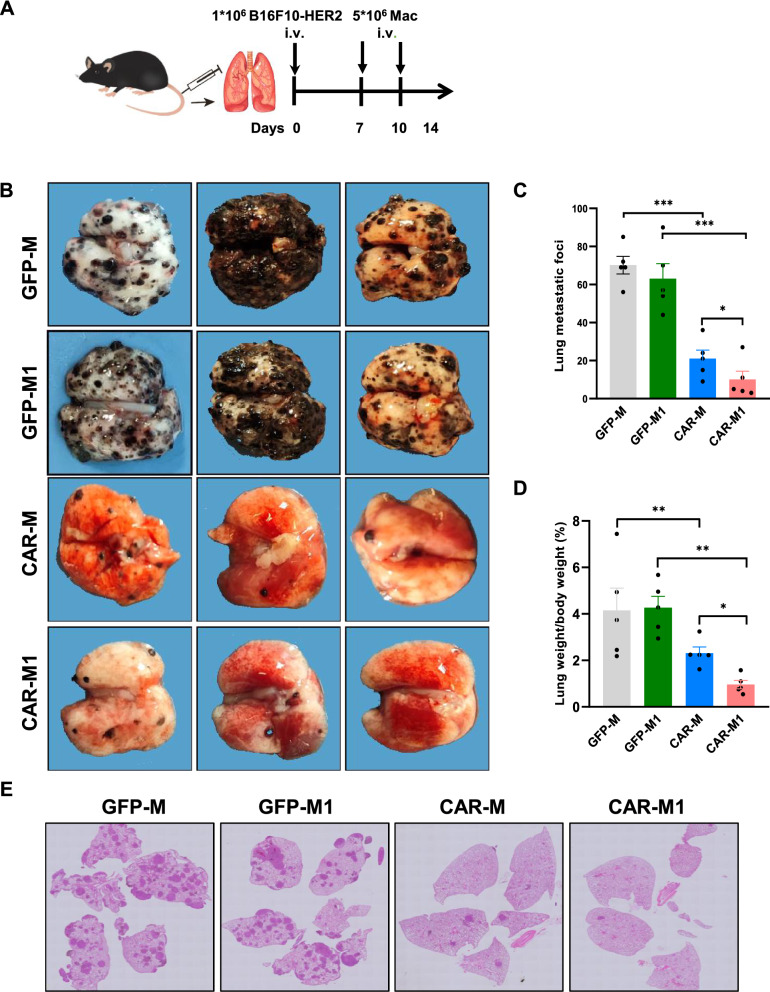
Systemic administration of M1-polarized CAR-Ms resulted in a potent antitumor response in a melanoma lung metastasis model. **A** An illustration of the experimental design. **B** Representative gross images of lungs excised from the indicated treatment groups at the experimental endpoint. **C** Quantitative analysis of metastatic foci from (**B**). Data are shown as the mean ± SEM. **D** Antitumoural efficacy was assessed by the lung weight-to-body weight ratio of mice bearing intravenous B16F10-HER2 cells posttreatment. **E** Lung metastatic burden assessed by HE staining. *, *p* < 0.05; **, *p* < 0.01; ***, *p* < 0.001

Previous studies have shown that macrophages accumulate in the lung after venous infusion. We established a syngeneic melanoma lung metastasis model in which human HER2-expressing B16F10-HER2 melanoma cells were implanted intravenously. On days 7 and 10 after tumor cell implantation, engineered macrophages were infused intravenously as shown in Fig. 6A. On day 14, the mice were sacrificed, and we found that the number of metastatic nodules in the lungs of mice treated with CAR-Ms and M1-polarized CAR-Ms was significantly reduced (Fig. 6B, C). In addition, we detected a significant reduction in the metastatic burden by evaluating the lung weight-to-body weight ratio (Fig. 6D). HE staining analysis also showed that the area of metastasis in the lung tissue was significantly reduced after CAR-M and CAR-M1 infusion (Fig. 6E). These results suggested that systemic administration of CAR-Ms could significantly reduce the metastatic tumor burden in the lung colonization model.

## Discussion

CARs are synthetic receptors expressed on immune cells via genetic engineering. They contain an extracellular antigen recognition domain derived from a single chain antibody variable fragment or a bound receptor/ligand, a hinge, a transmembrane domain, and an intracellular signaling activation domain that triggers immune cell activation [17]. CARs allow effector cells to specifically recognize target antigens and induce immune cells to exert cytotoxic effects. CARs were first used to arm T lymphocytes, and CAR-T therapies have achieved excellent efficacy in the treatment of certain hematologic malignancies, including B-cell-derived leukemia and lymphoma [18–20]. The success of CAR-T therapy for hematological malignancies has prompted the application of this approach in solid cancers. However, the dissemination of CAR-T cells in solid tumors has not been satisfactory. The main factors that limit the effects of CAR-T cells in solid tumors include poor trafficking and infiltration, an immunosuppressive tumor microenvironment, CAR-T exhaustion, and the antigen heterogeneity of solid tumors, which lead to poor killing function [21]. Researchers have programmed other immune cells with CAR, such as natural killer cells [22], gamma-delta T cells [23], and macrophages [8], aiming to enhance CAR-mediated therapeutic effects, and some of these candidates have shown potential to overcome some defects of CAR-T cells. However, continued exploration is required to identify better therapeutic candidates for solid tumors.

As professional antigen-presenting cells in the innate immune system, macrophages exert powerful phagocytic and cytotoxic effects, prime adaptive immune responses through antigen presentation, and secrete a series of proinflammatory cytokines and chemokines, which play a central role in defense against pathogens [6]. More importantly, macrophages are abundant in most solid tumors. They can efficiently penetrate and persist in solid tumor tissues, including breast and lung tumor tissues [24]. These characteristics make macrophages more suitable for the role of CAR carrier cells. Several CAR-M-based clinical trials have been approved by the FDA [25]. By introducing CAR into macrophages, specific CAR-mediated recognition further enhances macrophage phagocytosis, killing and antigen presentation. CAR-Ms enable epitope spreading and are able to overcome antigen escape in solid tumors [8]. In addition, CAR-Ms secrete proinflammatory cytokines and chemokines to improve the immunosuppressive TME and promote T-cell recruitment into the TME. All these properties suggest that CAR-Ms may have certain advantages over CAR-T cells in treating solid tumors.

To develop an efficient CAR structure for macrophages, we chose a humanized single-chain antibody targeting HER2 as the recognition region. The FcR common γ subunit, which can trigger phagocytosis and has often been used in other constructs [26–28], was fused to the C-terminus of the scFv (Fig. 1A). High transfection efficiency was achieved using a lentivirus transduction system (Fig. 1C, D). Our data suggested that the novel CAR-Ms we designed could efficiently phagocytose and kill HER2-positive tumor cells in vitro (Fig. 2).

Macrophages are highly heterogeneous immune cells that exhibit different polarization characteristics in response to various microenvironmental signals. Proinflammatory M1 and anti-inflammatory M2 macrophages coexist within the tumor microenvironment (TME). In most solid tumors, M2-like macrophages dominate tumor-associated macrophages (TAMs), and they support tumor growth, promote angiogenesis, facilitate invasion and metastasis, and mediate treatment resistance and immunosuppression [24]. As TAMs account for the majority of infiltrating immune cells and are highly plastic in the TME [29, 30], strategies have been developed to reeducate M2 macrophages to generate M1 macrophages. Blocking the CD47-SIRPα signaling axis and activating TLR or CD40 signaling have been demonstrated to promote TAM repolarization and have shown promising therapeutic effects in preclinical models [31]. Recent studies have shown that monophosphatidyl lipid A (MPLA) combined with IFN-γ can promote reprogramming of TAMs in situ [32]. Exosomes loaded with oligonucleotides targeting STAT6, a key transcription factor that regulates the polarization of M2-type macrophages, were delivered to tumor tissues to reprogram TAMs toward tumoricidal macrophages [33]. These studies suggest that reprogramming macrophages toward M1-like macrophages could induce potent antitumor therapeutic effects.

The therapeutic potential of a defined subpopulation of macrophages has also been explored extensively [34]. Rybalko et al. demonstrated that M1-polarized macrophages could promote muscle functional recovery and inhibit fibrotic tissue deposition [35]. Ma et al. demonstrated that M1-polarized macrophages ameliorated liver fibrosis in mice and exerted therapeutic effects by recruiting endogenous immune cells to modulate the immune microenvironment. Moreover, M1-polarized macrophages had a similar distribution and could maintain their polarized phenotypes for at least 14 days in vivo [36]. Michael et al. also demonstrated that the M1 phenotype could be maintained for at least 40 days after viral transduction [8]. These studies suggested that the administration of polarized macrophages might have a better therapeutic effect than the administration of unstimulated macrophages.

A key issue affecting the clinical application of CAR-M-based immunotherapy is how a proinflammatory phenotype of CAR-Ms can be acquired or strengthened within the immunosuppressive TME. Klichinsky et al. demonstrated that macrophages transduced with the replication-deficient adenovirus Ad5f35 exhibited an M1-like proinflammatory phenotype along with efficient CAR expression, which persisted and facilitated TME remodeling in vivo [8]. Fiber-substituted adenovirus serotype 5 vectors containing the fiber protein from adenovirus serotype 35 (Ad5f35) exhibit properties that render them suitable as a platform for targeted adenovirus vectors. Ad5F35 exhibits efficient transduction in a variety of human cells in vitro. In contrast, systemic administration of Ad5f35 vectors into mice mediates low levels of transduction efficiencies in organs. The refractoriness of mice to Ad5f35 vectors is due to the low expression of CD46, the receptor of Ad5f35, in mouse cells [16]. In this study, we used a lentivirus system to infect murine-derived macrophages. Our data demonstrated that macrophages did not exhibit an activation state similar to that observed after exposure to adenovirus after lentivirus transduction, indicating that lentivirus transduction itself did not directly induce a proinflammatory phenotype. Previous studies have shown that IFN-γ pretreatment enhanced the proinflammatory phenotype of macrophages and was well tolerated in cancer patients [37]. These studies suggest that CAR-Ms may further enhance cytotoxic antitumor activity after M1-type polarization.

Phagocytosis, cytokine and chemokine production, and antigen presentation function can be significantly enhanced after M1 polarization in vitro. We hypothesized that antitumor effects may be further enhanced after the administration of M1-polarized CAR-Ms. To polarize macrophages toward the M1 phenotype, we pretreated macrophages with LPS and IFN-γ before infusion. Our data showed that the phagocytosis of CAR-Ms was further enhanced after M1 polarization in vitro. M1-polarized CAR-Ms were more potent in killing cancer cells and could produce more proinflammatory cytokines. The in vivo antitumor effects of M1-polarized CAR-Ms were further confirmed in several murine tumor models, including primary tumor and metastatic tumor models by local or systemic injection models.

A critical limitation of the clinical application of CAR-T cells is cytokine release syndrome associated with CAR-T-cell expansion, which causes an inflammatory cytokine storm [38]. It is reasonable to have similar concerns about the application of CAR-Ms. However, although M1-type polarization induced high levels of proinflammatory cytokine production in vitro, we did not observe severe systemic cytotoxicity associated with CAR-M administration, indicating that CAR-M treatment is safe and well tolerated.

It should be noted that although M1-type polarization in vitro is a powerful approach, it induces a transient proinflammatory phenotype, and we were not able to measure how long the macrophages maintained their M1 polarization status in vivo. Therefore, it is essential to explore approaches to promote a more stable and durable M1-type polarization status to enhance CAR-M efficacy, as well as to investigate other approaches to optimize CAR-Ms and overcome known challenges, such as antigen heterogeneity in solid tumors [39]. In addition, the M1/M2 classification method we used to describe the polarization state of macrophages in our study is changing. In fact, macrophages are highly plastic immune cells, and their polarization is a dynamic and complex process. The simple M1/M2 polarization paradigm, especially in the in vivo microenvironment, is limited in describing macrophage phenotypes, and macrophage heterogeneity should be described according to its function [13]. However, the M1/M2 paradigm is still widely used in the literature, especially for inducing macrophages to display proinflammatory or antiinflammatory phenotypes. This classification is simple and effective, which is sufficient to meet the needs in our study.

In conclusion, our results showed that M1-polarized CAR-Ms exhibited enhanced antitumor efficacy and represent a promising approach for adoptive immune cell therapy.

## Supplementary Information

**Additional file 1:** **Fig. S1.** The purity of macrophages differentiated from bone marrow cells. **Fig. S2.** CAR-mediated phagocytosis in J774A.1 macrophages. **Fig. S3. **M1-polarized CAR-Ms induce killing effects in vitro. **Fig. S4. **Lentiviral transduction does not induce a proinflammatory phenotype in macrophages. **Fig. S5. **Evaluation of the side effects of M1-polarized CAR-M treatment. **Table S1.** The antibody resources used for flow cytometry analyses in the study.

## Data Availability

The original contributions presented in the study are included in the article/Additional file, and further inquiries can be directed to the corresponding author.
