# Supplementary material for: M1 polarization enhances the antitumor activity of chimeric antigen receptor macrophages in solid tumors
Source: J Transl Med. 2023 Mar 28;21:225. doi: 10.1186/s12967-023-04061-2 (PMC10044396; doi:10.1186/s12967-023-04061-2)
Supplement: Supplementary file 1 — Additional file 1: Fig. S1. The purity of macrophages differentiated from bone marrow cells. Fig. S2. CAR-mediated phagocytosis in J774A.1 macrophages. Fig. S3. M1-polarized CAR-Ms induce killing effects in vitro. Fig. S4. Lentiviral transduction does not induce a proinflammatory phenotype in macrophages. Fig. S5. Evaluation of the side effects of M1-polarized CAR-M treatment. Table S1. The antibody resources used for flow cytometry analyses in the study. [file 12967_2023_4061_MOESM1_ESM.docx]

Additional file

M1 polarization enhances the antitumor activity of chimeric antigen receptor macrophages in solid tumors

Yi Huo^1,2,3^^†^, Han Zhang ^2†^, Longqi Sa^4†^, Wenjing Zheng^2^, Yang He^5^, Haohan Lyu^2^, Mengjie Sun^2^, Lingling Zhang^2^, Lequn Shan^4^*, Angang Yang^1^* and Tao Wang ^2^*

^1^ State Key Laboratory of Cancer Biology, Department of Immunology, Air Force Medical University, Xi’an, Shaanxi, China

^2^ State Key Laboratory of Cancer Biology, Department of Medical Genetics and Developmental Biology, Air Force Medical University, Xi’an, Shaanxi, China

^3^ Department of Laboratory Medicine, 941 Hospital of Joint Logistics Support Force of PLA, Xining, Qinghai, China

^4^ Department of Spine Surgery, Honghui Hospital, Xi'an Jiaotong University, Xi'an, Shaanxi, China

^5^ The Second Clinical Medical College of Lanzhou University, Lanzhou University, Lanzhou, Gansu, China.

^†^These authors contributed equally.

*** Correspondence:**

Lequn Shan, email: drshanlq@fmmu.edu.cn

Angang Yang, email: agyang@fmmu.edu.cn

Tao Wang, email: wangt@fmmu.edu.cn

**
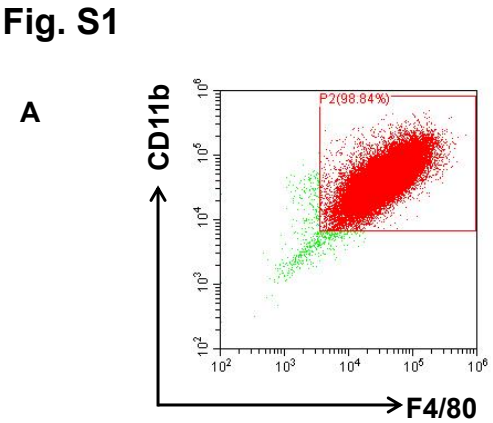
**

**Fig. S1 The purity of macrophages differentiated from bone marrow cells.**

Flow cytometry was used to analyze the percentage of BMDMs (shown as the surface marker F4/80^+^CD11b^+^ population) differentiated from C57BL/6 mice.

**
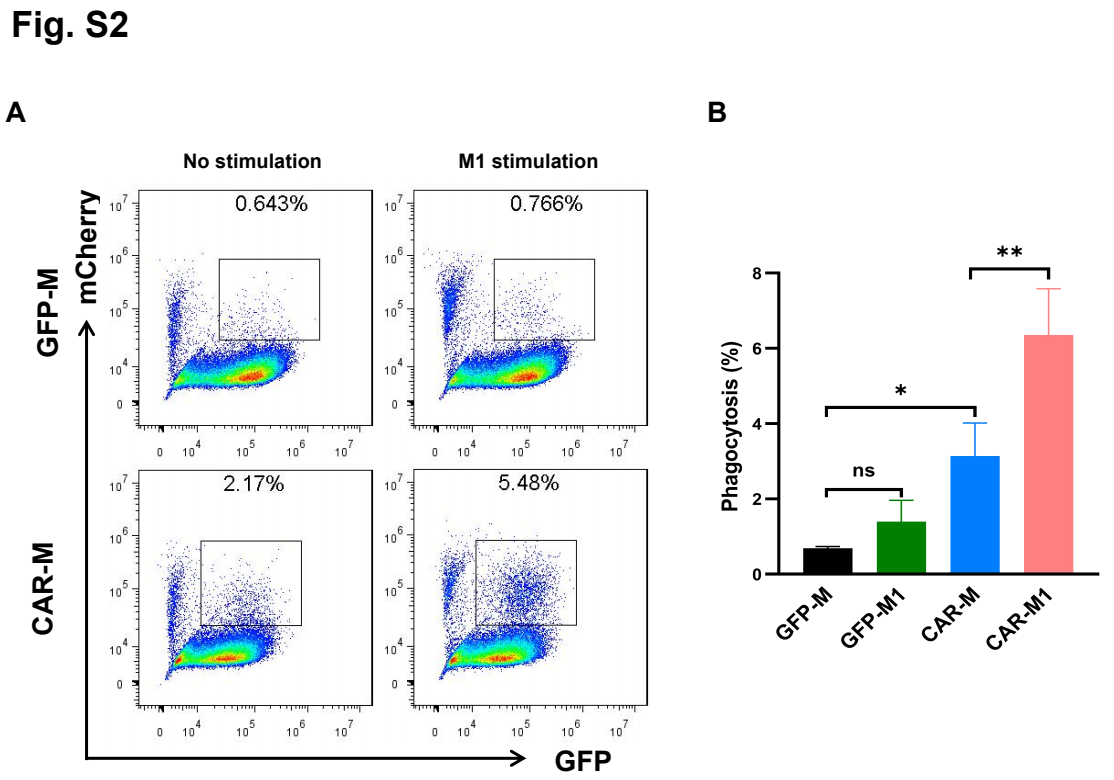
**

**Fig. S2 CAR-mediated phagocytosis in J774A.1 macrophages.**

**A** Representative flow cytometry assay showing phagocytosis of mCherry+MC38-HER2 cells by J774A.1 cells expressing GFP or CAR at an E:T=5:1 ratio. **B** Quantitative analysis of the percentage of phagocytosis by flow cytometry. Three independent experiments were performed. Data are shown as the mean ± SEM *, *p* < 0.05; **, *p* < 0.01; ***, *p* < 0.001.


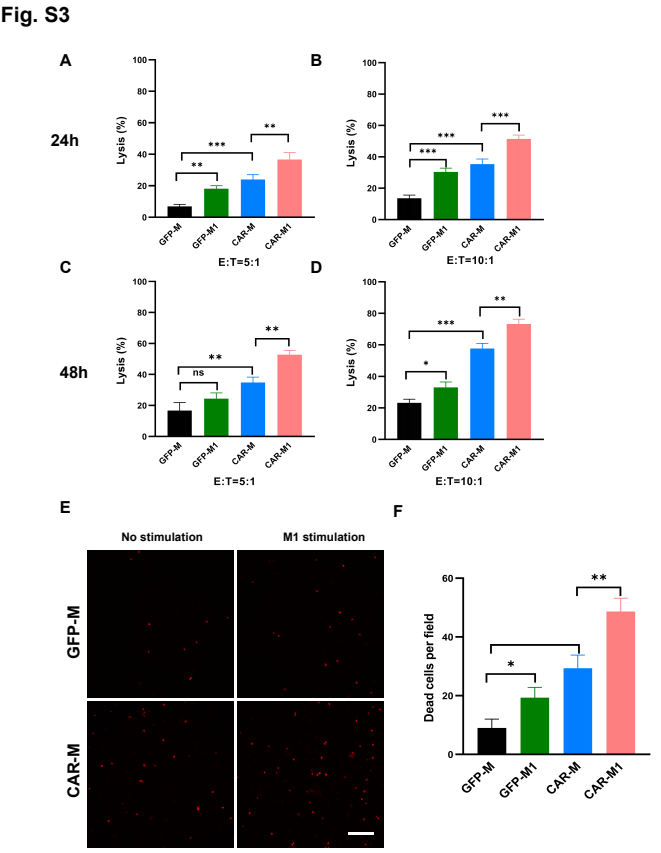


**Fig. S3 M1-polarized CAR-Ms induce killing effects *in vitro.***

**A**-**D** Killing potential was evaluated by a luciferase-based killing assay of Luc^+^MC38-HER2 cells by J774A.1 cells after 24 hours and 48 hours of coculture at different E:T ratios in vitro. Data represent the mean±s.d. of three independent replicates. **E** Representative fluorescence microscopic imaging showing the CAR-M-mediated killing effect assessed with the YoYo-3 far-red fluorescent dye in vitro, scale bar: 100 µm. **F** Statistical analysis of the data from **E**. Data are shown as the mean ± SEM. *, *p* < 0.05; **, *p* < 0.01; ***, *p* < 0.001.

**
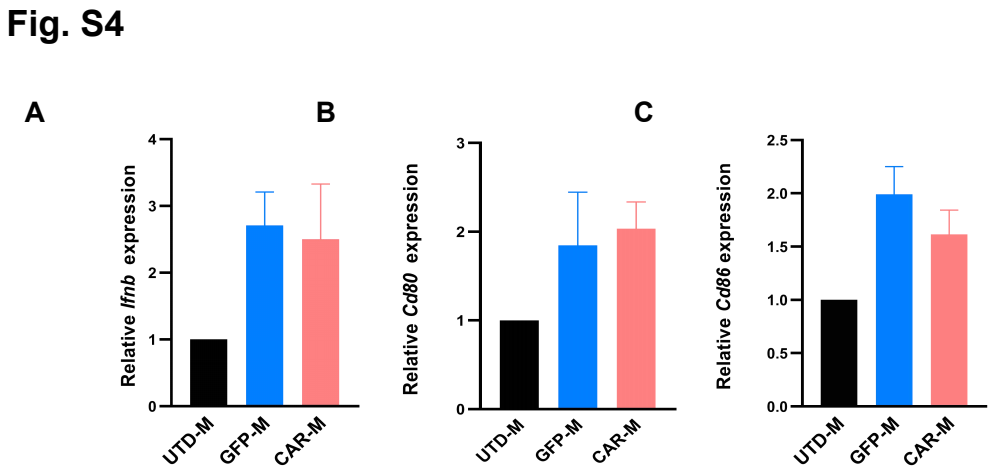
 Fig. S4 Lentiviral transduction does not induce a proinflammatory phenotype in macrophages.**

**A**-**C** Relative mRNA expression levels of Ifnb (**A**), Cd80 (**B**), and Cd86 (**C**) in UTD-Ms, GFP-Ms, and CAR-Ms by qRT‒PCR.

**
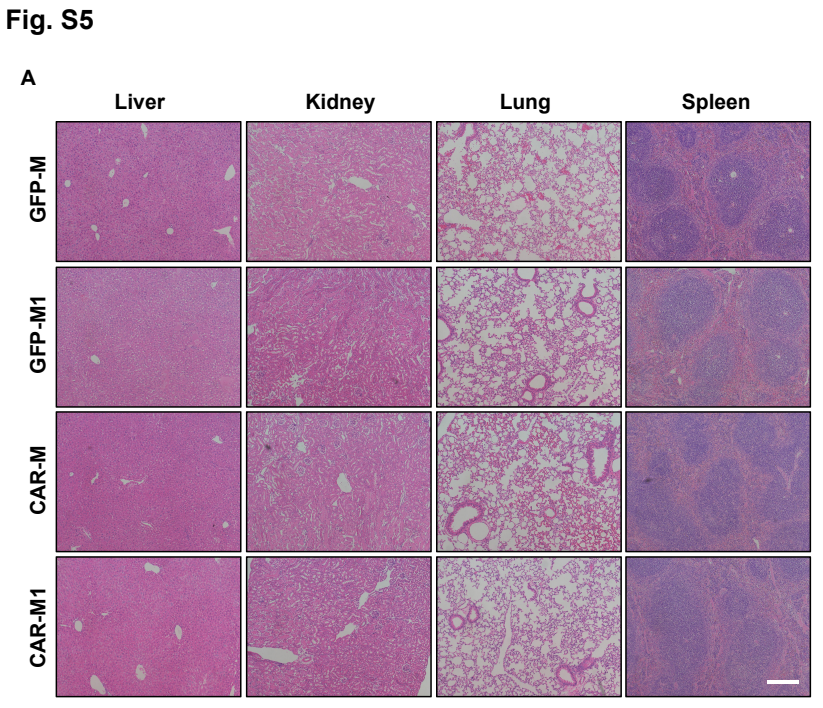
**

**Fig. S5 Evaluation of the side effects of M1-polarized CAR-M treatment.**

Representative hematoxylin and eosin (HE) staining of major organs, including the liver, lung, kidney, and spleen, from C57 mice bearing subcutaneous B16F10-HER2 melanoma tumors in the GFP-M, GFP-M1, CAR-M, and CAR-M1 treatment groups. Scale bar: 200 µm.

**Table S1**

| Antibody | Cat# | Source |
| --- | --- | --- |
| APC anti-human CD340 (erbB2/HER2) | 324407 | BioLegend |
| PerCP/Cyanine5.5 anti-mouse CD80 | 104721 | BioLegend |
| APC/Cyanine 7 anti-mouse CD86 | 105029 | BioLegend |
| PE anti-mouse CD11b | 101208 | BioLegend |
| APC anti-mouse F4/80 | 123116 | BioLegend |
| APC anti-His Tag | 362605 | BioLegend |
